# Association between conditioning intensity and height growth after allogeneic hematopoietic stem cell transplantation in children

**DOI:** 10.1002/cam4.6336

**Published:** 2023-07-11

**Authors:** Suguru Uemura, Daiichiro Hasegawa, Kenji Kishimoto, Tomoko Fujikawa, Sayaka Nakamura, Aiko Kozaki, Atsuro Saito, Toshiaki Ishida, Takeshi Mori, Kayo Ozaki, Yoshiyuki Kosaka

**Affiliations:** ^1^ Department of Hematology and Oncology Kobe Children's Hospital Kobe Japan; ^2^ Department of Endocrinology and Metabolism Kobe Children's Hospital Kobe Japan

**Keywords:** conditioning intensity, growth, hematopoietic stem cell transplantation, late effects, pediatrics

## Abstract

**Background:**

The present study aimed to examine the association between the conditioning intensity and height growth in pediatric patients who underwent allogeneic hematopoietic stem cell transplantation (allo‐HSCT).

**Methods:**

We reviewed the clinical records of 89 children with malignant diseases who underwent initial allo‐HSCT between 2003 and 2021. Height measurements were standardized using standard height charts prepared by the Japanese Society for Pediatric Endocrinology to calculate standard deviation score (SDS). We defined short stature as a height SDS less than −2.0 in that reference. Myeloablative conditioning (MAC) comprised total‐body irradiation at more than 8 Gy and busulfan administration at more than 8 mg/kg (more than 280 mg/m^2^). Other conditioning regimens were defined as reduced intensity conditioning (RIC).

**Results:**

A total of 58 patients underwent allo‐HSCT with MAC, and 31 patients received allo‐HSCT with RIC. There were significant differences in the height SDS at 2 and 3 years after allo‐HSCT between MAC and RIC group (−1.33 ± 1.20 vs. −0.76 ± 1.12, *p* = 0.047, −1.55 ± 1.28 vs. −0.75 ± 1.11, *p* = 0.022, respectively). Multivariate logistic regression analysis with the adjustments for potential confounding factors of patients less than 10 years of age at allo‐HSCT and chronic graft‐versus host disease demonstrated that MAC regimen was associated with a markedly increased risk of a short stature at 3 years after allo‐HSCT (adjusted odds ratio, 5.61; 95% confidence interval, 1.07–29.4; *p* = 0.041).

**Conclusion:**

The intensity of conditioning regimen may be associated with short statures after allo‐HSCT.

## Graphical abstract

There were significant differences in the height SDS at 2 and 3 years after allo‐HSCT between MAC and RIC group (−1.33 ± 1.20 vs. −0.76 ± 1.12, *p* = 0.048, −1.55 ± 1.28 vs. −0.75 ± 1.11, *p* = 0.022, respectively).

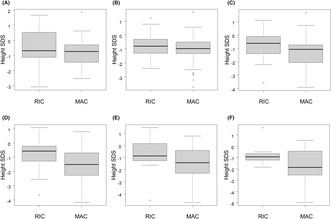

## 1 INTRODUCTION

Due to the improvements in therapies for cancer in childhood, including chemotherapy, surgery, and radiation, exceeding 80% of children who were diagnosed with cancer now become long‐term survivors.^1^, ^2^, ^3^ However, long‐term survivors often experience late effect complications related with their health, behavior, and quality of life (QOL), that require medical care even after the completion of the treatment for cancer.^3^, ^4^, ^5^

Allogeneic hematopoietic stem cell transplantation (allo‐HSCT) is one of the effective therapies for high‐risk hematological diseases, some solid tumors, and primary immunodeficiency.^6^ High‐dose chemotherapy and total body irradiation (TBI), which have been illustrated to increase the antitumor effect, are often used as conditioning regimens for allo‐HSCT.^7^ However, late effects of childhood cancer survivors (CCS) who underwent allo‐HSCT are at increased risk of late effects, compared to patients treated without allo‐HSCT.^8^, ^9^, ^10^ Although long‐term survival is expected by many pediatric patients who receive allo‐HSCT, they have a substantially increased burden of serious late effects.^11^, ^12^

Growth disorder is one of the most common late effects of allo‐HSCT in children and often influences the final adult height among CCS.^13^, ^14^ Growth disorder in patients who have received allo‐HSCT is caused by the growth hormone (GH) deficiency, which is induced by the irradiation to the pituitary‐hypothalamus system.^15^ The irradiation to the body is also associated with impaired bone growth.^16^ The irradiation as a conditioning regimen or systemic steroids for the treatment of graft‐versus host disease (GVHD) directly inhibit the bone growth.^17^ Previous reports revealed that growth disorder was associated with disease type, age at allo‐HSCT, and total dose of irradiation, and GVHD.^13^, ^14^, ^15^, ^16^, ^17^, ^18^ Therefore, there was a need for the establishment of the optimal strategy for allo‐HSCT to reduce the endocrine complications and improve the QOL of CCS who have received allo‐HSCT.

Reduced intensity conditioning (RIC) regimens represent a well‐recognized and established approach for allo‐HSCT for malignant and non‐malignant diseases in both children and adults.^19^, ^20^, ^21^ The aim of RIC is to decrease short‐ and long‐term complications induced by myeloablative conditioning (MAC) with promoting adequate engraftment and stable donor‐derived hematopoiesis.^22^, ^23^ However, few previous reports have investigated the effect of conditioning intensity on growth after allo‐HSCT in children.^24^

In the present study, we examined the association between the conditioning intensity and height growth in the pediatric patients who received allo‐HSCT.

## 2 PATIENTS AND METHODS

### 2.1 Study population

In this study, a total of 120 consecutive patients who underwent initial allo‐HSCT for malignant disease at our institute between April 1, 2003, and December 31, 2021 were included. Patients who relapsed (*n* = 11) or died (*n* = 9) within 1 year of allo‐HSCT, or experienced primary graft failure (*n* = 10) were excluded. A patient with trisomy 21 was also excluded because of the innate short stature compared to patients without trisomy 21. Finally, data on 89 patients were analyzed retrospectively. We reviewed the medical records to obtain the patients' characteristics including clinical information. The last clinical follow‐up date in the present study was on December 31, 2022.

The present study was approved by the ethics review committees of Kobe Children's Hospital (R4‐142).

### 2.2 Conditioning regimen

The conditioning regimen was determined by the physician's intent considering patient's disease, disease status, and donor type. MAC comprised TBI at more than 8 Gy and busulfan administration at more than 8 mg/kg (more than 280 mg/m^2^).^25^ Other conditioning regimens were defined as RIC.

### 2.3 Height evaluations

Height was routinely measured at the time of allo‐HSCT and then annually until the age of 18 years or final height, as part of medical examinations. Final height was defined as the tallest height measured at age 18 years or older, and when height velocity was inferior to 1 cm per year for consecutive 2 years. Endocrinological disorders after allo‐HSCT were also extracted from medical records. The height standard deviation score (SDS) was calculated using the growth charts with the reference to Japanese children's age and sex prepared by the Japanese Society for Pediatric Endocrinology. The growth charts consisted of the datasets obtained from the Japanese national survey in the year 2000, including 18,550 anthropometric measurements of babies and infants surveyed by the Ministry of Health, Labour and Welfare and 695,600 anthropometric measurements of school children surveyed by the Ministry of Education, Culture, Sports, Science, and Technology, as established by the lambda‐mu‐sigma method.^26^, ^27^

Short stature was defined as a height SDS less than −2.0 for individuals of the same sex and chronologic age in that reference.

### 2.4 Statistical analysis

In our study, the primary outcome measure was the height SDS. The secondary outcome measure was the development of short stature. The patients who relapsed or died of any cause more than 1 year after allo‐HSCT were censored. The chi‐squared or Fisher's exact test was used to compare categorical variables in patients' characteristics between the MAC and RIC groups. The unpaired *t*‐test or Mann–Whitney *U* test was also used to compare continuous variables in patients' characteristics between the MAC and RIC groups. By Spearman's rank correlation test, the correlation between height SDS and TBI dose or age at allo‐HSCT was assessed. A multivariate logistic regression model was used to calculate the adjusted odds ratio (OR) for short stature at 3 years after allo‐HSCT with a 95% confidence interval (CI). The development of chronic GVHD (cGVHD) or systemic steroids for the treatment of cGVHD, and age less than 10 years at allo‐HSCT were included in the model as potential confounding factors. For all models, the number of examined covariates was determined according to the number of outcome events, with 10 events required for one covariate. Reported *p*‐values of less than 0.05 were considered statistically significant. All statistical analyses were conducted using EZR (Saitama Medical Center, Jichi Medical University), which is a modified version of R commander (The R Foundation for Statistical Computing).^28^

## 3 RESULTS

### 3.1 Clinical characteristics

The median follow‐up period after allo‐HSCT was 2338 days (range, 375–6835 days). Median age at allo‐HSCT was 8.3 years (range, 0.5–16.2 years). The primary diagnoses were as follows: acute lymphoblastic leukemia (ALL, *n* = 39), acute myeloid leukemia (AMZL, *n* = 20), myelodysplastic syndrome/myeloproliferative disease (*n* = 17), malignant lymphoma (*n* = 4), and solid tumors (*n* = 9) (Table 1). ALL was the most common primary disease in the MAC group (58.6%), but not in the RIC group (16.1%). There was a significant difference between the MAC and RIC groups in the distribution of the primary disease (*p* < 0.001). There was also a significant difference in donor source between the MAC and RIC groups (*p* = 0.041). There were no statistically significant differences in gender, donor type, HLA compatibility, use of TBI, craniospinal irradiation (CSI) prior to allo‐HSCT, acute GVHD, chronic GVHD (cGVHD), or use of systemic steroids for the treatment of cGVHD between the MAC and RIC groups. Six patients, all in the MAC group, were treated with GH or thyroid hormone (TH) replacement after allo‐HSCT. However, there was no significant difference in the number of patients who were treated with GH or TH replacement after allo‐HSCT between the MAC and RIC groups (*p* = 0.088).

**TABLE 1:** Clinical characteristics of the studied cohort.

Characteristics	MAC	RIC	*p* value
Number of patients	58	31	
Median age at allo‐HSCT, years (range)	8.1 (0.5–16.2)	9.8 (1.5–16.2)	0.15
Age at allo‐HSCT ≥10 years, *n* (%)	39 (67.2)	17 (54.8)	0.26
Gender, *n* (%)			0.19
Female	23 (39.7)	17 (54.8)	
Male	35 (60.3)	14 (45.2)	
Disease, *n* (%)			<0.001
ALL	34 (58.6)	5 (16.1)	
AML	10 (17.2)	10 ((32.3)	
MDS/MPD	6 (10.4)	11 (35.5)	
ML	2 (3.4)	2 (6.4)	
Solid tumor	6 (10.4)	3 (9.7)	
Donor source, *n* (%)			0.041
Bone marrow	36 (62.1)	27 (87.1)	
Peripheral blood	4 (6.9)	0 (0.0)	
Cord blood	18 (31.0)	4 (12.9)	
Donor type, *n* (%)			1.00
Related	17 (29.3)	9 (29.0)	
Unrelated	41 (70.7)	22 (71.0)	
HLA compatibility, *n* (%)			0.13
Matched	13 (36.1)	66 (51.2)	
Mismatched	23 (63.9)	63 (48.8)	
Remission status at allo‐HSCT in acute leukemia, *n* (%)			0.64
CR1	24 (54.5)	10 (66.7)	
CR2	16 (36.4)	5 (33.3)	
Non‐CR	4 (9.1)	0 (0.0)	
Use of TBI, *n* (%)	40 (69.0)	24 (77.4)	0.47
CSI prior to allo‐HSCT, *n* (%)	3 (5.2)	0 (0.0)	0.55
GVHD prophylaxis, *n* (%)			0.097
Tacrolimus based	43 (74.1)	28 (90.3)	
Cyclosporine based	15 (25.9)	3 (29.7)	
aGVHD, *n* (%)			
Any	40 (69.0)	18 (58.1)	0.35
Grade II–IV	27 (46.6)	15 (48.4)	1.00
cGVHD, *n* (%)	19 (32.8)	10 (32.3)	1.00
Systemic steroids for the treatment of cGVHD, *n* (%)	8 (13.8)	4 (12.9)	1.00
GH/TH replacement, *n* (%)	6 (10.3)	0 (0.0)	0.088

Abbreviations: ALL, acute lymphoblastic leukemia; allo‐HSCT, allogeneic hematopoietic stem cell transplantation; AML, acute myeloid leukemia; CR, complete remission; GH, growth hormone; GVHD, graft versus host disease; HLA, human leukocyte antigen; MAC, myeloablative conditioning; MDS, myeloid dysplastic syndrome; ML, malignant lymphoma; MPD, myeloid proliferative disease; RIC, reduced intensity conditioning; TBI, total body irradiation; TH, thyroid hormone.

### 3.2 Annual changes in height SDS after allo‐HSCT

**FIGURE 1:**
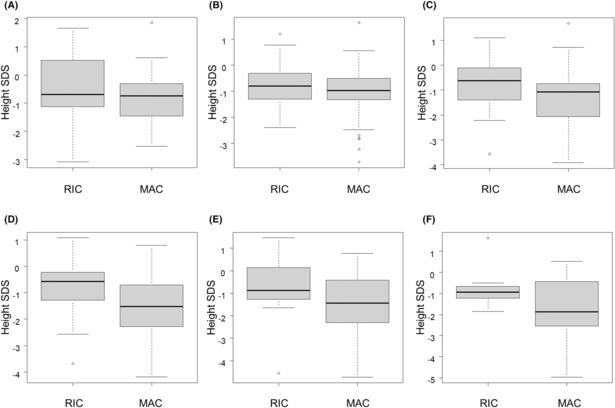
Annual height SDS at and after allo‐HSCT. A, Height SDS at allo‐HSCT. −0.81 ± 0.88 in the MAC group and −0.54 ± 1.12 in the RIC group (*p* = 0.22). B, Height SDS 1 year after allo‐HSCT. −1.08 ± 1.02 in the MAC group and −0.77 ± 0.85 in the RIC group (*p* = 0.19). C, Height SDS 2 year after allo‐HSCT. −1.33 ± 1.20 in the MAC group and −0.76 ± 1.12 in the RIC group (*p* = 0.047). D, Height SDS 3 year after allo‐HSCT. −1.55 ± 1.28 in the MAC group and −0.75 ± 1.11 in the RIC group (*p* = 0.022). E, Height SDS 4 year after allo‐HSCT. −1.59 ± 1.47 in the MAC group and −0.80 ± 1.35 in the RIC group (*p* = 0.080). F, Height SDS 5 year after allo‐HSCT. −1.67 ± 1.57 in the MAC group and −0.79 ± 0.90 in the RIC group (*p* = 0.094). allo‐HSCT, allogeneic hematopoietic stem cell transplantation; MAC, myeloablative conditioning; RIC, reduced intensity conditioning; SDS, standard deviation score.

Figure 1 shows the height SDS on allo‐HSCT, and 1, 2, 3, 4, and 5 years after allo‐HSCT. The height SDS in the MAC group on allo‐HSCT and 1, 2, 3, 4, and 5 years after allo‐HSCT were −0.81 ± 0.88, −1.08 ± 1.02, −1.33 ± 1.20, −1.55 ± 1.28, −1.59 ± 1.47, and −1.67 ± 1.57, respectively (Figure 1), whereas the values in the RIC group were −0.54 ± 1.12, −0.77 ± 0.85, −0.76 ± 1.12, −0.75 ± 1.11, −0.80 ± 1.35, and − 0.79 ± 0.90, respectively (Figure 1). A significant reduction in the height SDS at 2 and 3 years after allo‐HSCT in MAC group was observed, compared with those in RIC group (*p* = 0.047 and 0.022, respectively) (Figure 1C,D).

The comparison between male and female patients revealed no significant differences in height SDS on allo‐HSCT, and 1, 2, 3, 4, and 5 years after allo‐HSCT (Table S1).

Patients with and without ALL were also compared, because corticosteroids are key drugs for ALL and often suppress GH secretion and the function of insulin‐like growth factor (IGF)‐I and other factors.^29^ No significant differences were found between the ALL and non‐ALL groups in height SDS on allo‐HSCT, and 1, 2, 3, 4, and 5 years after allo‐HSCT (Table S2).

### 3.3 Effects of age at allo‐HSCT and TBI dose on height SDS

Table 2 shows the correlation between the height SDS at 2 and 3 years after allo‐HSCT and age at allo‐HSCT. There was no statistically significant correlation between the height SDS at 2 and 3 years after allo‐HSCT and age at allo‐HSCT and (*r* = 0.030, *p* = 0.81 and *r* = 0.027, *p* = 0.85, respectively).

**TABLE 2:** Correlation coefficient to height SDS at 2 and 3 years after allo‐HSCT.

Height SDS at 2 years after allo‐HSCT		
Characteristics	Correlation coefficient	*p* value
Age	0.030	0.81
TBI dose	0.13	0.23

Height SDS at 3 years after allo‐HSCT		
Characteristics	Correlation coefficient	*p* value
Age	0.027	0.85
TBI dose	0.020	0.88

Abbreviations: allo‐HSCT, allogeneic hematopoietic stem cell transplantation; SDS, standard deviation score; TBI, total body irradiation.

The correlation between the height SDS at 2 and 3 years after allo‐HSCT and TBI dose are also shown in Table 2. The height SDS at 2 and 3 years after allo‐HSCT did not show statistically significant correlations with total doses of TBI (*r* = 0.13, *p* = 0.23 and *r* = 0.020, *p* = 0.88, respectively).

### 3.4 Association between conditioning intensity and the development of short stature

Table 3 demonstrates the results of logistic regression analyses to investigate adjusted OR of short stature at 3 years after allo‐HSCT. The MAC regimen was associated with a marked increase in risk of the short stature at 3 years after allo‐HSCT (adjusted OR, 5.61; 95% CI, 1.07–29.4). Age less than 10 years at allo‐HSCT was not associated with an increased risk of the short stature at 3 years after allo‐HSCT (adjusted OR, 0.93; 95% CI, 0.25–3.55). The development of cGVHD was not associated with an increased risk of the short stature at 3 years after allo‐HSCT (adjusted OR, 1.14; 95% CI, 0.27–4.76). The use of systemic steroids for the treatment of cGVHD demonstrated no association with an increased risk of short stature 3 years after allo‐HSCT (adjusted OR, 1.77; 95% CI, 0.25–12.5) (Table S3).

**TABLE 3:** Multivariate logistic regression analysis for the development of short stature at 3 years after allo‐HSCT.

	Crude OR	95% CI	*p* value	Adjusted OR	95% CI	*p* value
RIC	Ref					
MAC	5.43	1.07–27.4	0.041	5.61	1.07–29.4	0.041
No cGVHD	Ref					
cGVHD	0.83	0.22–3.16	0.79	1.14	0.27–4.76	0.86
≥ 10 years of age at allo‐HSCT	Ref					
< 10 years of age at allo‐HSCT	1.08	0.30–3.83	0.91	0.93	0.25–3.55	0.92

Abbreviations: allo‐HSCT, hematopoietic stem cell transplantation; cGVHD, chronic graft‐versus host disease; CI, confidence interval; MAC, myeloablative conditioning; OR, odds ratio; RIC, reduced intensity conditioning.

### 3.5 The effect of TBI as MAC on height SDS

**FIGURE 2:**
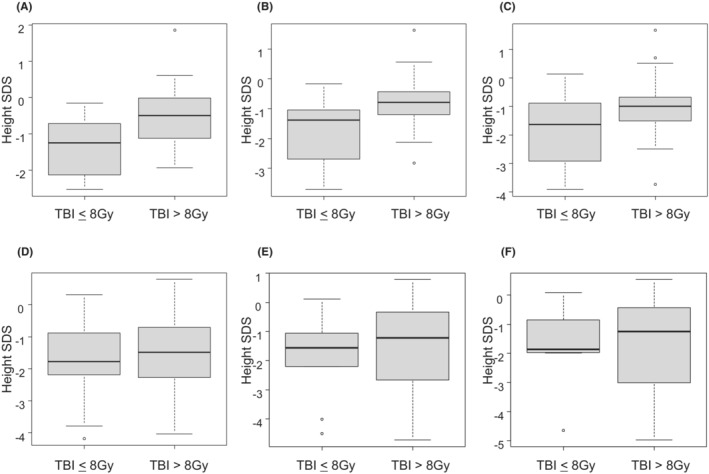
Annual height SDS at and after allo‐HSCT in the MAC group. A, Height SDS at allo‐HSCT. −0.56 ± 0.81 in the TBI >8 Gy group and −1.39 ± 0.79 in the TBI ≤8 Gy (*p* = 0.001). B, Height SDS 1 year after allo‐HSCT: −0.77 ± 0.82 and −1.78 ± 1.11 in the group with TBI of >8 and ≤8 Gy, respectively (*p* = 0.002). C, Height SDS 2 years after allo‐HSCT: −1.04 ± 1.07 and −1.85 ± 1.27 in the group with TBI of >8 and ≤8 Gy, respectively (*p* = 0.032). D, Height SDS 3 years after allo‐HSCT: −1.43 ± 1.21 and −1.72 ± 1.42 in the group with TBI of >8 and ≤8 Gy, respectively (*p* = 0.52). E, Height SDS 4 years after allo‐HSCT: −1.47 ± 1.51 and −1.85 ± 1.45 in the group with TBI of >8 and ≤8 Gy, respectively (*p* = 0.51). F, Height SDS 5 years after allo‐HSCT: −1.65 ± 1.62 and −1.73 ± 1.56 in the group with TBI of >8 and ≤8 Gy, respectively (*p* = 0.91). allo‐HSCT, allogeneic hematopoietic stem cell transplantation; SDS, standard deviation score; TBI, total body irradiation.

Table 4 shows the clinical characteristics of the 58 patients in the MAC group. The median age at allo‐HSCT of patients in the MAC group who received >8 Gy of TBI was 9.3 years (range, 1.8–16.2 years), whereas that of the patients who received ≤8 Gy of TBI was 2.0 years (range, 6 months–14.9 years). A significant difference was observed in age at allo‐HSCT between these two groups (*p* = 0.005). The height SDS in the group with TBI of >8 Gy on allo‐HSCT and 1, 2, 3, 4, and 5 years after allo‐HSCT were − 0.56 ± 0.81, −0.77 ± 0.82, −1.04 ± 1.07, −1.43 ± 1.21, −1.47 ± 1.51, and −1.65 ± 1.62, respectively (Figure 2), whereas that in the group with TBI of ≤8 Gy were −1.39 ± 0.79, −1.78 ± 1.11, −1.85 ± 1.27, −1.72 ± 1.42, −1.85 ± 1.45, and −1.73 ± 1.56, respectively (Figure 2). A significant reduction in the height SDS on allo‐HSCT, one and 2 years after allo‐HSCT in the group with TBI of ≤8 Gy, compared with the group with TBI of >8 Gy (*p* = 0.001, 0.002, and 0.032, respectively) (Figure 2A–C).

**TABLE 4:** Clinical characteristics of the studied cohort in the MAC group.

Characteristics	TBI > 8Gy	TBI ≤ 8Gy	*p* value
Number of patients	40	18	
Median age at allo‐HSCT, years (range)	9.3 (1.8–16.2)	2.0 (0.5–14.9)	0.005
Age at allo‐HSCT ≥10 years, *n* (%)	16 (40.0)	3 (16.7)	0.13
Gender, *n* (%)			0.385
Female	14 (35.0)	9 (50.0)	
Male	26 (65.0)	9 (50.0)	
Disease, *n* (%)			0.096
ALL	27 (67.5)	7 (38.9)	
AML	6 (15.0)	4 (22.2)	
MDS/MPD	2 (5.0)	4 (22.2)	
ML	2 (5.0)	0 (0.0)	
Solid tumor	3 (7.5)	3 (16.7)	
Donor source, *n* (%)			0.004
Bone marrow	29 (72.5)	7 (38.9)	
Peripheral blood	4 (10.0)	0 (0.0)	
Cord blood	7 (17.5)	11 (61.1)	
Donor type, *n* (%)			0.061
Related	15 (37.5)	2 (11.1)	
Unrelated	25 (62.5)	16 (88.9)	
HLA compatibility, *n* (%)			0.57
Matched	18 (45.0)	6 (33.3)	
Mismatched	22 (55.0)	12 (66.7)	
Remission status at allo‐HSCT in acute leukemia, *n* (%)			0.022
CR1	14 (42.4)	10 (90.9)	
CR2	15 (45.4)	1 (9.1)	
Non‐CR	4 (12.2)	0 (0.0)	
CSI prior to allo‐HSCT, *n* (%)	3 (7.5)	0 (0.0)	0.55
GVHD prophylaxis, *n* (%)			0.34
Tacrolimus based	27 (67.5)	15 (83.3)	
Cyclosporine based	13 (32.5)	3 (16.7)	
aGVHD, *n* (%)			
Any	29 (72.5)	11 (61.1)	0.54
Grade II–IV	20 (50.0)	7 (38.9)	0.57
cGVHD, *n* (%)	14 (35.0)	5 (27.8)	0.76
Systemic steroids for cGVHD, *n* (%)	5 (12.5)	3(16.7)	0.699
GH/TH replacement, *n* (%)	4 (10.0)	2 (11.1)	1.00

Abbreviations: ALL, acute lymphoblastic leukemia; allo‐HSCT, allogeneic hematopoietic stem cell transplantation; AML, acute myeloid leukemia; CR, complete remission; GH, growth hormone; GVHD, graft versus host disease; HLA, human leukocyte antigen; MAC, myeloablative conditioning; MDS, myeloid dysplastic syndrome; ML, malignant lymphoma; MPD, myeloid proliferative disease; TBI, total body irradiation; TH, thyroid hormone.

## 4 DISCUSSION

Herein, we demonstrate that MAC regimen increased the risk of a short stature after allo‐HSCT in the present study. Conditioning intensity may be associated with growth disorder for pediatric patients after allo‐HSCT. Few studies investigated the effect of conditioning intensity on short stature.^24^ Myers, et al. reported that the even RIC regimen was associated with significant late endocrine effects in children and young adults.^24^ This discrepancy with our results may be attributable to the cohort bias. Most subjects in the study by Myers et al. had non‐malignant diseases including immune disorders, metabolic, and genetic disorders, in contrast to the present study. Furthermore, some cases of these disorders are associated with short stature.^24^

Although there were significant differences in the height SDS at 2 and 3 years after allo‐HSCT between the MAC group and RIC group, the significant differences in the height SDS at 4 and 5 years after allo‐HSCT between the two groups were not observed. This result may be attributable to the effect of small number of patients with height measurements available at 4 and 5 years after allo‐HSCT.

Growth disorder after TBI as a conditioning regimen has been well documented.^15^, ^30^, ^31^ Patients who receive the irradiation to central nervous system (CNS) prior to allo‐HSCT develop in GH deficiency more frequently than those who do not receive irradiation to the CNS.^15^, ^31^ In our cohort, three patients received CSI. One of these patients underwent CSI followed by allo‐HSCT at 7 years of age with MAC regimen containing TBI at a dose of 12 Gy and required the GH therapy after allo‐HSCT.

Couto‐Silva et al. found that the prevalence of short stature was greater in the patients who received TBI at a younger age.^32^ Patients who received TBI before 4 years of age developed skeletal lesions leading to substantial height loss, despite GH treatment.^32^ The previous study comparing 12 Gy of TBI against a total Bu dose 16 mg/kg or 480 mg/m^2^ as a conditioning regimen after allo‐HSCT revealed that the patients who received TBI showed a significant decrease in their height SDS compared to Bu.^30^ In the present study, there were no correlations between height SDS and age at allo‐HSCT or the total dose of TBI. In the MAC group, median age at allo‐HSCT of patients who received >8 Gy of TBI was 9.3 years (range, 1.8–16.2 years), while the median age at allo‐HSCT among the patients who received ≤8 Gy of TBI was 2.0 years (range, 6 months–14.9 years). Between the two MAC regimens, there was a significant difference in age at allo‐HSCT (*p* = 0.005). This was predominantly due to physician's bias such as the avoidance of TBI containing regimens in children who underwent allo‐HSCT at a younger age. As a matter of fact, a significant reduction was observed in the height SDS at allo‐HSCT in the group with TBI of <8 Gy (Figure 2).

Corticosteroids are key drugs for ALL and often suppress GH secretion and the function of IGF‐I and other factors.^29^ Moreover, corticosteroids are used for the treatment of cGVHD and prolonged use is expected to be at high risk for growth. However, there were no significant differences between the ALL and non‐ALL groups in height SDS on allo‐HSCT, and 1, 2, 3, 4, and 5 years after allo‐HSCT in the present study (Table S2). The use of systemic steroids for the treatment of cGVHD revealed no association with an increased risk of short stature 3 years after allo‐HSCT (Table S3). A previous report by Sanders et al revealed that patients with ALL or Non‐Hodgkin lymphoma (NHL) had lower average height SDS at the diagnosis of GH deficiency and were less effective for GH replacement therapy, compared with non‐ALL such as AML.^14^ However, the proportion of patients who received cranial irradiation before allo‐HSCT was significantly higher in ALL or NHL than in others. Another report from European Bone Marrow Transplantation Group on the final height of patients who received allo‐HSCT revealed no significant difference was in the delta‐SDS, which is defined the difference between the height SDS at allo‐HSCT and that at the final height between the ALL, AML, or chronic myeloid leukemia groups.^33^ Further studies are needed to identify the risk of steroids for the longitudinal height growth of the patients with ALL who received allo‐HSCT following chemotherapy, including steroids.

Some previous reports revealed male gender as one of the factors associated with decreased the height SDS.^14^, ^33^ However, our cohort demonstrated no significant differences between males and females in height SDS on allo‐HSCT, and 1, 2, 3, 4, and 5 years after allo‐HSCT (Table S1). The small sample size might affect the difference between our results and that of the previous reports.

The present study had some limitations. First, due to the nature of retrospective analysis, we were unable to completely rule out the physician's bias such as the avoidance of TBI containing regimens or MAC regimens for patients who received allo‐HSCT at a younger age. Second, because of the heterogeneity in the primary diseases and the significant difference in the primary diseases between the MAC and RIC groups, the treatments for different primary diagnoses provided prior to allo‐HSCT might affect height growth. Third, many patients in our cohort did not reach their final heights before the end of the study period. In the present study, median age at allo‐HSCT was 8.3 years with 7.3 years of median follow‐up period. Therefore, longer follow‐up periods are required to determine the effect of condition intensity on height growth after allo‐HSCT in children.

In conclusion, conditioning intensity may be associated with a short stature after allo‐HSCT in children. Further studies are needed to validate this observed association between conditioning intensity and height growth of children after allo‐HSCT.

## AUTHOR CONTRIBUTIONS

**Suguru Uemura:** Conceptualization (lead); data curation (lead); formal analysis (lead); investigation (lead); methodology (lead); project administration (lead); software (lead); writing – original draft (lead); writing – review and editing (lead). **Daiichiro Hasegawa:** Conceptualization (supporting); formal analysis (supporting); investigation (supporting); methodology (supporting); project administration (supporting); software (supporting); supervision (lead); writing – original draft (supporting); writing – review and editing (supporting). **Kenji Kishimoto:** Data curation (supporting); formal analysis (supporting); investigation (supporting); methodology (supporting); writing – review and editing (supporting). **Tomoko Fujikawa:** Data curation (supporting); formal analysis (supporting); investigation (supporting); methodology (supporting); project administration (supporting); writing – original draft (supporting). **Sayaka Nakamura:** Data curation (supporting); investigation (supporting); project administration (supporting); writing – review and editing (supporting). **Aiko Kozaki:** Conceptualization (supporting); data curation (supporting); investigation (supporting); project administration (supporting). **Atsuro Saito:** Data curation (supporting); investigation (supporting); project administration (supporting); writing – review and editing (supporting). **Toshiaki Ishida:** Data curation (supporting); investigation (supporting); project administration (supporting); writing – review and editing (supporting). **Takeshi Mori:** Data curation (supporting); investigation (supporting); project administration (supporting); writing – review and editing (supporting). **Kayo Ozaki:** Data curation (supporting); formal analysis (supporting); investigation (supporting); methodology (supporting); project administration (supporting); writing – review and editing (supporting). **Yoshiyuki Kosaka:** Conceptualization (supporting); data curation (supporting); methodology (supporting); project administration (supporting); supervision (lead); writing – review and editing (supporting).

## FUNDING INFORMATION

No funding was received.

## CONFLICT OF INTEREST STATEMENT

The authors declare no conflict of interest.

## ETHICS STATEMENT

This study was approved by the Ethics Committee of Kobe Children's Hospital (R4‐142). Because the present study was retrospectively analyzed, informed consent, or assent was waived and opt‐out system was presented.

## Supporting information


Table S1.
Table S2.
Table S3.

## Data Availability

The data that support the findings of this study are available from the corresponding author, upon reasonable request.
