# Supplementary material for: Association between conditioning intensity and height growth after allogeneic hematopoietic stem cell transplantation in children
Source: Cancer Med. 2023 Jul 11;12(16):17018–27. doi: 10.1002/cam4.6336 (PMC10501226; doi:10.1002/cam4.6336)
Supplement: Supplementary file 1 — Table S1. Table S2. Table S3. [file CAM4-12-17018-s001.docx]

Table S1, Annual changes in height SDS after allo-HSCT according to gender

| At time | Height SDS in male | Height SDS in female | P value |
| --- | --- | --- | --- |
| On allo-HSCT | −0.62 ± 1.00 | −0.82 ± 0.95 | 0.36 |
| 1 year after | −0.91 ± 1.03 | −0.99 ± 0.89 | 0.72 |
| 2 years after | −1.08 ± 1.20 | −1.14 ± 1.13 | 0.82 |
| 3 years after | −1.22 ± 1.23 | −1.27 ± 1.33 | 0.88 |
| 4 years after | −1.34 ± 1.52 | −1.29 ± 1.44 | 0.91 |
| 5 years after | −1.28 ± 1.49 | −1.49 ± 1.42 | 0.68 |

Table S2, Annual changes in height SDS after allo-HSCT according to disease type

| At time | Height SDS in ALL | Height SDS in non-ALL | P value |
| --- | --- | --- | --- |
| On allo-HSCT | −0.62 ± 0.91 | −0.78 ± 1.03 | 0.48 |
| 1 year after | −0.80 ± 0.83 | −1.06 ± 1.04 | 0.25 |
| 2 years after | −0.84 ± 1.06 | −1.33 ± 1.20 | 0.080 |
| 3 years after | −1.00 ± 1.03 | −1.43 ± 1.41 | 0.22 |
| 4 years after | −1.07 ± 1.25 | −1.53 ± 1.62 | 0.30 |
| 5 years after | −1.29 ± 1.34 | −1.49 ± 1.57 | 0.70 |

Table S3, Multivariate logistic regression analysis for the development of short stature at three years after allo-HSCT

|  | Crude OR | 95% CI | P value |  | Adjusted OR | 95% CI | P value |
| --- | --- | --- | --- | --- | --- | --- | --- |
| RIC | Ref |  |  |  |  |  |  |
| MAC | 5.43 | 1.07-27.4 | 0.041 |  | 5.94 | 1.11-31.6 | 0.037 |
|  |  |  |  |  |  |  |  |
| No use of systemic steroids for the treatment of cGVHD | Ref |  |  |  |  |  |  |
| Use of systemic steroids for the treatment of cGVHD | 0.83 | 0.22-3.16 | 0.79 |  | 1.77 | 0.25-12.5 | 0.57 |
|  |  |  |  |  |  |  |  |
| > 10 years of age at allo-HSCT | Ref |  |  |  |  |  |  |
| < 10 years of age at allo-HSCT | 1.08 | 0.30-3.83 | 0.91 |  | 0.88 | 0.23-3.42 | 0.86 |
